# The genetic diversity and structure of *indica* rice in China as detected by single nucleotide polymorphism analysis

**DOI:** 10.1186/s12863-016-0361-x

**Published:** 2016-03-15

**Authors:** Qun Xu, Xiaoping Yuan, Shan Wang, Yue Feng, Hanyong Yu, Yiping Wang, Yaolong Yang, Xinghua Wei, Ximing Li

**Affiliations:** State Key Laboratory of Rice Biology, China National Rice Research Institute, Hangzhou, 310006 China

**Keywords:** Single nucleotide polymorphism (SNP), Rice (*Oryza sativa* L.), Genetic diversity, Structure, Array

## Abstract

**Background:**

Rice (*Oryza sativa* L.) is the staple food of more than half of the world’s population. The identification of genetic diversity in local varieties of rice compared with that of improved or introduced varieties is important in breeding elite varieties for sustainable agriculture. Array-based single nucleotide polymorphism (SNP) detection is a useful technique for such studies and breeding applications.

**Results:**

We developed a 5291-SNP genome-wide array and used it to genotype 471 *indica* rice accessions in China using Illumina’s Infinium technology. Local, introduced, and improved rice varieties were clustered into three sub-groups, with some overlapping shown in principal component analysis and neighbor-joining tree, also confirmed by model-based structure. A minor allele frequency ≥0.2 was observed in 72 % of polymorphic SNPs in local rice varieties, which was higher than that in other sub-groups. Local rice varieties also had the highest mean polymorphism information content (PIC) and genetic diversity. Analysis of molecular variance showed that 90.61 % of genetic variation was a result of differences within sub-groups.

**Conclusions:**

Our results revealed that SNP analysis clustered local varieties, introduced varieties, and improved varieties into three clear sub-groups. The distribution of parameter PIC values on sub-group genomes revealed that genetic differentiation among them might not be on a genome-wide scale, but rather on selected loci or chromosomal intervals. The result of Gene Ontology enrichment analysis showed that genes nearby those selected SNPs associated different molecular functions or various traits among sub-groups.

**Electronic supplementary material:**

The online version of this article (doi:10.1186/s12863-016-0361-x) contains supplementary material, which is available to authorized users.

## Background

According to the International Rice Research Institute, rice (*Oryza sativa* L.) is the staple food for more than 3.5 billion people worldwide, which represents around half of the world’s population. Importantly, rice is the staple food in China and throughout Asia. Tracing back the history of rice breeding in China, several different stages of development can be observed: (1) the beginning of systematic rice breeding in 1919; (2) the use of pure line selection as the most common method of breeding from the 1920s to the 1940s; and (3) extensive national cooperation in rice breeding involving various effective breeding methods, such as the use of local varieties as parents, and the introduction of foreign varieties after 1949 [1].

Local varieties of rice have evolved from their wild progenitors under both natural and human selection, resulting in a high level of genetic diversity [2]. Therefore, they represent the main sources with which to undertake genetic improvement for the toleration of biotic and abiotic stress. Moreover, identifying the genetic diversity of local varieties compared with improved or introduced varieties will assist the breeding of elite varieties for use in sustainable agriculture.

Single nucleotide polymorphisms (SNPs) are rapidly replacing simple sequence repeats (SSRs) as the DNA marker of choice for applications in plant breeding and genetics [3]. Many studies have investigated the genomic structure and genetic diversity of rice based on second-generation sequencing technologies, and have identified millions of SNPs [2, 4–6]. Currently, array-based SNP detection is one of the major high-throughput marker detection platforms and can be used to genotype multiple samples within a short period, providing data that are straightforward to analyze [7, 8].

Based on the genotyping of 471 *indica* rice accessions using 5291 SNPs distributed throughout all 12 chromosomes, the objectives of this study were: 1) to analyze the population structure and compare the level of diversity between local, introduced, and improved varieties of rice; and 2) to investigate genetic differentiation between these three subgroups.

## Results

### Characteristics of SNPs

**Fig. 1:**
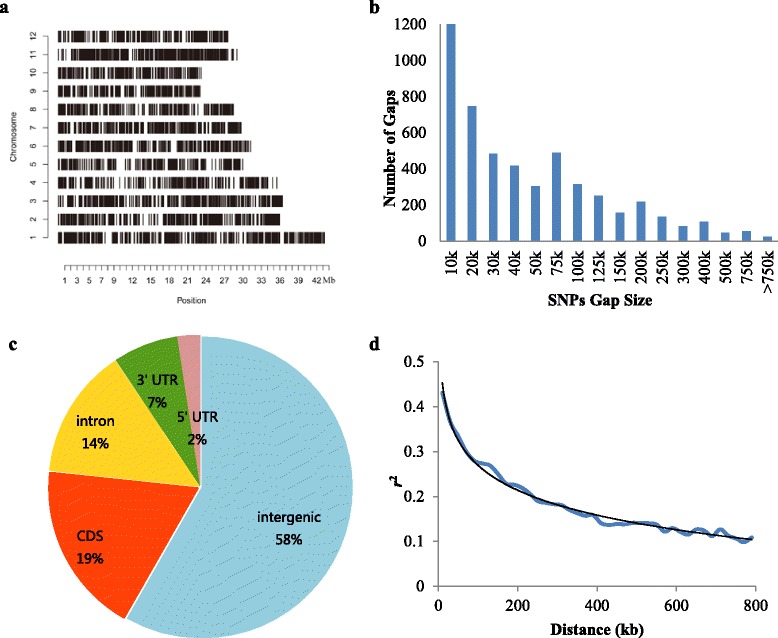
Characteristics of SNPs and LD decay. **a** SNP distribution along the entire rice genome. **b** Distances between the SNPs. **c** Localization of SNPs at the whole genome level. **d** LD decay estimated from 471 *indica* rice

Out of a total of 5291 SNPs, 5060 were successfully used to evaluate accessions based on GenTrain scores and missing rates (Additional file 1: Table S1). These SNPs are distributed evenly throughout the entire rice genome (Fig. 1a). A histogram of the inter-SNP spacing distribution generated by mapping SNPs onto the rice MSU6.0 genome assembly (Fig. 1b) showed that the gaps between 72.33 % of SNPs are less than 75 kb, and that all gaps exceed 0.5 kb. Most SNPs are located in intergenic regions, while 42 % are located in genic regions (Fig. 1c) including untranslated regions, introns, and coding sequences. A regression analysis between *r*^*2*^ and genetic distance indicated that they fitted the equation *y* = −0.08*ln*(x) + 0.6377 (*R*^2^ = 0.9875) (Fig. 1d). The genome-wide LD decay distance was ~196.08 kb at which the *r*^*2*^ dropped to half its maximum value.

### Genetic structure of *indica* rice

**Fig. 2:**
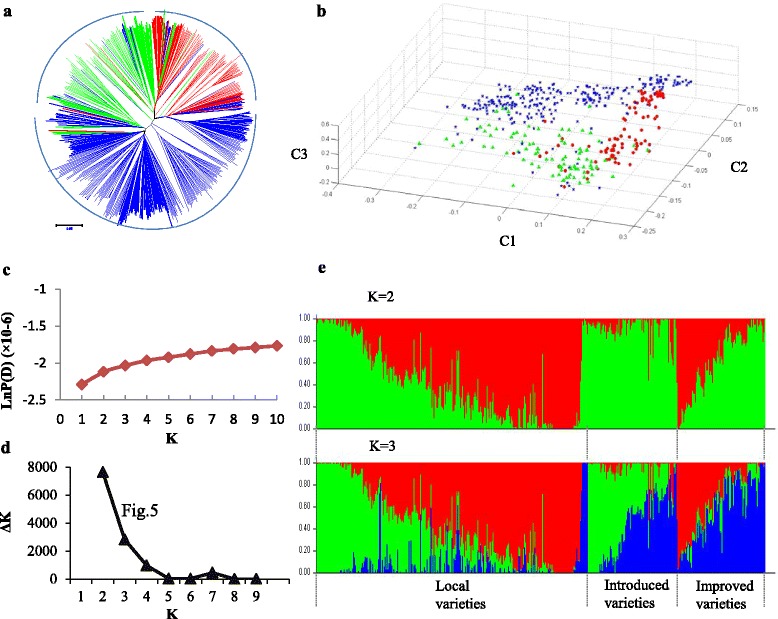
Population structure of 471 *indica* rice based on 5060 SNPs. **a** PCA of *indica* rice. Local, introduced, and improved varieties are colored *blue*, *red*, and *green* in **a**-**b**. **b** Neighbor-joining tree of *indica* rice constructed from a simple matching distance. **c** Mean LnP(D) values plotted as the number of subpopulations. **d** ΔK values plotted as the number of subpopulations. **e** Subpopulations (K = 2 and K = 3) inferred using STRUCTURE

Principal component analysis (PCA) indicated that local, introduced, and improved varieties of rice could be clustered into three distinct sub-groups (Fig. 2b). However, some overlapping among these groups were observed. In particular, improved varieties widely overlapped with other sub-groups, probably reflecting the fact that breeding activities led to genetic similarities.

The neighbor-joining clustering method tree representing relationships based on Nei’s genetic distance also showed that the 471 *indica* rice accessions clustered together into three sub-groups with some overlapping (Fig. 2a).

The model-based simulation of population structure showed that the LnP(D) value for each given K, from 1 to 10, increased but did not show a maximum value (Fig. 2c). The value of Evanno’s ΔK showed the highest peak at K = 2, then at K = 3 (Fig. 2d). When K = 3, the result (Fig. 2e) was in accordance with the clustered results showed in PCA and neighbor-joining tree.

### Genetic characterization within the three sub-groups

Basic statistics of whole genome SNP genetic diversity among local, introduced, and improved variety sub-groups of rice are listed in Table 1. Of the 5060 SNPs, 88.2 % were polymorphic in local varieties of rice, while 86.8 and 89.3 % were polymorphic in introduced and improved varieties, respectively. Over all chromosomes, SNP polymorphism levels ranged from 79 to 96 % in local varieties, from 80 to 93 % in introduced varieties, and from 81 to 96 % in improved varieties (Additional file 2: Table S2).

**Table 1:** Comparison of polymorphism information among rice sub-groups

	Local varieties	Introduced varieties	Improved varieties
Monomorphic	597	667	539
Polymorphic	4463	4393	4521
Observed heterozygosity (H_O_)	0.0122	0.0080	0.0101
Gene diversity	0.3212	0.2868	0.3106
PIC	0.2533	0.2285	0.2465
MAF <0.05	1032	1239	1064
MAF 0.05–0.1	152	405	248
MAF 0.1–0.2	681	723	758
MAF ≥0.2	3195	2693	2990

The observed heterozygosity (H_O_) of almost all SNPs (>99 %) was lower than 0.2 in each sub-group, with an average of 0.01 (Table 1). In local varieties of rice, 72 % of polymorphic SNPs had a minor allele frequency (MAF) ≥0.2, while 10 % had a MAF <0.05; 61 % of polymorphic SNPs in introduced varieties had a MAF ≥0.2, with 13 % <0.05; while 66 % of polymorphic SNPs in improved varieties had a MAF ≥0.2, with 12 % <0.05. Both gene diversity (GD) and the average PIC of local varieties of rice were higher than in introduced and improved varieties. A maximum PIC value of 0.375 was found for all three sub-groups.

**Fig. 3:**
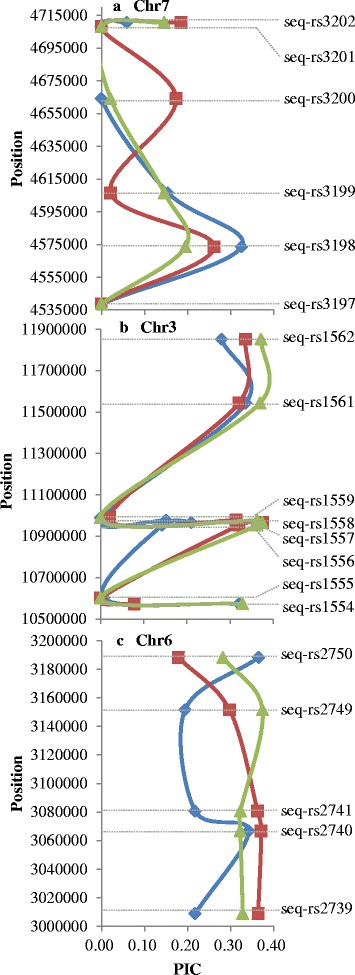
Examples of parameter PIC value distribution on genomes. *Blue*, *red*, and *green curves* show PIC changes in local, introduced, and improved varieties, respectively

Figure 3 shows examples of genetic diversity with parameter PIC values distributed on genomes among local, introduced, and improved variety sub-groups. We found that genetic differentiation between the sub-groups is not on a genome-wide scale, but rather on selected loci or chromosome intervals. Table 2 lists PIC values among sub-groups of SNPs used in Fig. 3, and their corresponding genes identified by BLAST search using SNP flanking sequences as a query. A total of 11 (58 %) SNPs are located in genes annotated in the MSU Rice Genome Annotation Project (6.0), of which four were labeled as cloned in the China Rice Data Center.

**Table 2:** PIC value among sub-groups of SNPs used in Fig. 3 and their genes identified by BLAST

SNP name	Chr.	Position	MSU locus	Cloned	PIC		
					Local varieties	Introduced varieties	Improved varieties
seq-rs1556	3	10952279	LOC_Os03g19480	*SDG718*	0.1423	0.3201	0.3629
seq-rs1557	3	10965826	LOC_Os03g19510	*OsClpP5*	0.2103	0.3750	0.3739
seq-rs1558	3	10976468	LOC_Os03g19520	/	0.1521	0.3142	0.3629
seq-rs2740	6	3066751	LOC_Os06g06540	/	0.3423	0.3716	0.3233
seq-rs2741	6	3080670	LOC_Os06g06560	*OsSSI*	0.2179	0.3632	0.3233
seq-rs2750	6	3188215	LOC_Os06g06770	/	0.3655	0.1788	0.2824
seq-rs3198	7	4573557	LOC_Os07g08840	*OsTRXh1*	0.3250	0.2617	0.1948
seq-rs3199	7	4606495	LOC_Os07g08880	/	0.1544	0.0208	0.1462
seq-rs3200	7	4664150	LOC_Os07g08970	/	0.0000	0.1735	0.0213
seq-rs3201	7	4708187	LOC_Os07g09020	/	0.0000	0.0000	0.0000
seq-rs3202	7	4710622	LOC_Os07g09020	/	0.0593	0.1853	0.1462

The population differentiation statistics (*F*_*ST*_) between local and introduced variety sub-groups was estimated at 0.1340, as well as at 0.0894 between local and improved variety sub-groups, and at 0.0871 between introduced and improved variety sub-groups (Table 3). It indicated a stronger differentiation between local varieties and introduced varieties than other pairwise sub-groups.

**Table 3:** The population differentiation statistics (*F*_*ST*_) between pairwise sub-groups

	Local varieties	Introduced varieties	Improved varieties
Introduced varieties	0.1340	/	/
Improved varieties	0.0894	0.0871	/

Analysis of molecular variance (AMOVA) among local, introduced, and improved variety sub-groups according to genome revealed a highly significant level of variation (*P* <0.0001; Table 4). Of this, 9.39 % reflected differences among sub-groups, and the remaining 90.61 % reflected differences within sub-groups.

**Table 4:** Analysis of molecular variance (AMOVA) of sub-group effect

Source	*df*	Variance component	Percentage of variance (%)	*P*
Among sub-groups	2	84.47	9.39	<0.0001
Within sub-groups	939	815.54	90.61	/
Total	941	900.01	/	/

## Discussion

Many crop species can be assessed using high density SNP genotyping arrays given sufficient sequence information of high quality [7–11]. Herein, we developed a 5291-SNP genome-wide array based on Illumina’s Infinium technology and used it to genotype 471 *indica* rice accessions in China.

Our array offers some advantages over other rice arrays. First, the SNPs in this array were selected from re-sequencing data of 950 rice accessions, while the SNPs in the Affymetrix 44 K array reported by Zhao et al. [12] were selected from the *Oryza*SNP project that only used sequence information from 20 accessions [13], and the SNPs in the Rice SNP50 array reported by Chen et al. [7] were selected from 801 rice accessions. Second, 80 % (4048) of SNPs from this array showed MAF ≥0.05 in *indica* subspecies, while this was observed for only 55 % (20,259) and 69 % (35,427) of SNPs from Affymetrix 44 K and Rice SNP50 arrays, respectively. Moreover, 58 % of SNPs in the current array are located in intergenic regions compared with 42 % from the Rice SNP50 array, indicating that our array is more suited to the analysis of genetic diversity.

In our paper, the whole genome-wide was ~196.08 kb, which is in agreement with previous studies that cultivated rice had a long-range LD decay distance from ~100 to ~200 kb [2, 13].

To examine genetic population structure, we conducted PCA and constructed a neighbor-joining tree and model-based structure based on 5060 SNPs. We observed clustering of local, introduced, and improved varieties of rice into three sub-groups with overlapping among them, suggestive of breeding activities. The history of rice breeding in China shows that new varieties were usually selected from landraces during the early period, later from crosses between landraces and introduced varieties, and more recently from crosses between improved varieties [14]. This is similar to the breeding history of Chinese bread wheat [15]. Therefore, in our paper a stronger population differentiation between local varieties and introduced varieties was observed than that between local varieties and improved varieties or between introduced varieties and improved varieties.

GD and PIC findings indicated that the local varieties of rice had higher levels of genetic diversity than both introduced and improved varieties. This agrees with the study by Zhang et al. [16], who used SSR markers in *indica* rice in China, and with the work of Ram et al. [17] in India. Similarly, Chapman et al. [18] used SNPs to show that genetic diversity was highest among wild lines of sunflowers and other crops and lowest among improved lines, with primitive domesticates being intermediate. The PIC value inferred by SNPs in the present study (0.3745) was notably lower than those of 0.52 and 0.595 revealed by Garris et al. [19] and Wang et al. [20] using SSRs respectively, but was close to the 0.257 reported by Chen et al. [21] using SNPs.

PIC distribution of SNPs among sub-groups on chromosomes suggested that particular important loci or chromosomal intervals rather than whole genomes (or chromosomes) were responsible for the observed differences in SNP genetic diversity across genomes. This is consistent with findings in rice reported by Wei et al. [22] and in wheat reported by Hao et al. [15]. The Gene Ontology (GO) enrichment analysis of genes nearby those SNPs identified some overrepresented GO entries (Additional file 3: Table S3). GO items in which identified genes were enriched also associated different molecular functions or various traits among different sub-groups. For example, the PIC distribution of loci seq-rs3198 on chromosome 7 (Fig. 3a) reflects the higher genetic diversity of local varieties than of introduced/improved varieties. LOC_Os07g08840 nearby seq-rs3198 was annotated with overrepresented GO item (GO:0019725) with description of ‘cell homeostasis’ and GO:0008152 with description of ‘metabolic process’. All homeostatic control depends on tuning metabolic reaction within cell according to environments. Previous study found that this gene (*OsTRXh1*) encoding a subgroup I h-type Trx in rice which regulates the redox state of the apoplast and influences plant development and stress responses [23]. Redox state is reflected in the balance of several sets of metabolites whose inter conversion is dependent on their ratios. The inter conversion is necessary for launch and stop the response according to signal status corresponding to environment stimulates, such as stress. This implies the relationship between those identified genes and environment adaption and indicates that local varieties of rice have a more extensive genetic basis for adaptation at this location. By contrast, the PIC distribution of the interval from loci seq-rs1556 to seq-rs1558 on chromosome 3 (Fig. 3b) reflects a lower genetic diversity of local varieties than of introduced/improved varieties of rice. A gene nearby by seq-rs1556, LOC_Os03g19480, was annotated with overrepresented GO: 0009791 with description of ‘post-embryonic development’, GO:0040029 with description of ‘regulation of gene expression, epigenetic’, GO:0000003 with description of ‘reproduction’, and so on. This gene (*SDG718*) was previously cloned as rice an enhancer of zeste [E(z)], encoding the polycomb repressive complex2 (PRC2) key subunit together with *SDG711*, and the PRC2-mediated epigenetic repression of gene expression was shown to be involved in the accurate photoperiod control of rice flowering [24].

As well as seq-rs1556, seq-rs1557 showed higher diversity in introduced/improved rice. A nearby gene, LOC_Os03g19510 was annotated with overrepresented GO items, including GO:0009536 with description of ‘plastid’ and GO:0008152 with description of ‘metabolic process’. Previous study has found this gene as a chloroplast protease gene, *OsClpP5* [25]. LOC_Os06g06560 nearby seq-rs2741 on chromosome 6 (Fig. 3c) was annotated with overrepresented GO items including GO:0009536 with description of ‘plastid’, GO:0008152 with description of ‘metabolic process’ and GO:0009058 with description of ‘biosynthetic process’. The function of this gene (*OsSSI*) has been previously confirmed to encode starch synthase I, which affects the structure of amylopectin chains in endosperm and the gelatinization temperature [26]. This could be explained in two ways. First, the introduced varieties of rice have a wider geographical distribution than local varieties. Second, introduced/improved varieties have undergone artificial selection pressure to facilitate human preferences for cooking and eating.

## Conclusions

The present study genotyped 471 *indica* rice accessions in China using a 5291-SNP genome-wide array, which revealed that local, introduced, and improved varieties of rice could be clearly clustered into three sub-groups and that local varieties have the highest genetic diversity. Parameter PIC value distribution on the genomes of the three sub-groups suggested that genetic differentiation is not on a genome-wide scale, but rather on selected loci or chromosome intervals. The GO enrichment analysis of genes nearby selected loci revealed that those SNPs were associated with different molecular functions or various traits among different sub-groups.

## Methods

### Plant materials

A total of 471 *indica* rice accessions were used: 285 local varieties, 94 introduced varieties, and 92 improved varieties, which are shown in the Additional file 4: Table S4.

### SNP marker analysis

The 5291 SNP targets were selected from whole-genome re-sequencing data [2, 4] following these criteria: (1) only those SNPs with two alleles; (2) no other SNPs or InDels within 60 bp flanking regions of the SNP site on both sides; and (3) high GenTrain scores. SNP genotyping was conducted on the Illumina platform following the Infinium® HD Assay Ultra Protocol (Illumina, Inc. San Diego, CA). SNP alleles were called using the GenomeStudio Genotyping Module v1.8.4 (Illumina).

Out of 5291 SNPs, 3787 located within a 1 kb region and with aliases starting with “LOC_Os” coordinates were found by BLAST analysis of the MSU6.0 annotation of the genome of Nipponbare [ftp://ftp.plantbiology.msu.edu/pub/data/Eukaryotic_Projects/o_sativa/annotation_dbs/pseudomolecules/version_6.0/all.dir/]. These SNPs were then associated with cloned genes in the Rice Data Center [http://www.ricedata.cn/gene/], and 187 SNPs were found to be cloned or reported.

### Data analysis

We conducted individual level PCA using NTSYS-pc v2.1 software [27] to investigate the population structure. PIC, GD, MAF, *F*_*ST*_, and a neighbor-joining tree construction based on Nei’s genetic distance between pairwise individuals were performed by PowerMarker v3.25 [28]. A model-based cluster analysis was performed using the program STRUCTURE v2.2 [29, 30]. The optimum number of populations (*K*) was selected by testing for *K* = 1 to *K* = 10 using five independent runs of 10,000 burn-in runs followed by 100,000 iterations with a model allowing for admixture and correlated allele frequencies. Both the value of log likelihood [LnP(D)] and an *ad hoc* statistic ΔK [31] were used to reveal the suitable population structure. H_O_ and genetic variation within and among different populations were evaluated using AMOVA implemented in Arlequin suite v3.5 [32]. The LD parameter (*r*^*2*^) was computed using TASSEL v4.0 [33] to evaluate the level of LD between linked SNPs. The LD decay rate was measured as the chromosomal distance at which the average pairwise correlation coefficient dropped to half of its maximum value [2]. GO enrichment analysis was conducted using Fisher Exact test based on hypergeometric distribution.

## Availability of supporting data

All relevant data are available within the manuscript and its additional files.

## Additional files

Additional file 1: Table S1. Information on the 5060 SNPs. (XLSX 666 kb) Additional file 2: Table S2. Polymorphism among sub-groups at the chromosome level. (PDF 30 kb) Additional file 3: Table S3. GO enrichment analysis of genes listed in Table 2. (XLSX 11 kb) Additional file 4: Table S4. Origination and classification of 471 germplasm accessions. (XLSX 36 kb)

## Abbreviations

AMOVA: analysis of molecular variance
*F*_*ST*_: the population differentiation statistics
GO: Gene Ontology
H_O_: observed heterozygosity
LD: linkage disequilibrium
MAF: minor allele frequency
PCA: principal component analysis
PIC: polymorphism information content
PRC2: polycomb repressive complex2
SNP: single nucleotide polymorphism
SSRs: simple sequence repeats
