# Supplementary material for: The genetic diversity and structure of indica rice in China as detected by single nucleotide polymorphism analysis
Source: BMC Genet. 2016 Mar 15;17:53. doi: 10.1186/s12863-016-0361-x (PMC4793538; doi:10.1186/s12863-016-0361-x)
Supplement: Additional file 2: Table S2. — Polymorphism among sub-groups at the chromosome level. (PDF 30 kb) [file 12863_2016_361_MOESM2_ESM.pdf]

Table S2 Polymorphism among sub-groups at the chromosome level

Chromosome	Total	Local varieties		Introduced varieties		Improved varieties	
		Mono	Poly	Mono	Poly	Mono	Poly
1	592	79	513	85	507	58	534
2	555	61	494	67	488	56	499
3	441	57	384	60	381	28	413
4	397	36	361	43	354	19	378
5	268	39	229	43	225	10	258
6	393	47	346	61	332	67	326
7	408	79	329	84	324	69	339
8	373	52	321	53	320	36	337
9	300	34	266	42	258	35	265
10	264	55	209	54	210	51	213
11	681	29	652	46	635	86	595
12	388	29	359	29	359	24	364
